# Supplementary material for: Domain Walls Conductivity in Hybrid Organometallic Perovskites and Their Essential Role in CH3NH3PbI3 Solar Cell High Performance
Source: Sci Rep. 2015 Jun 19;5:11467. doi: 10.1038/srep11467 (PMC4473534; doi:10.1038/srep11467)
Supplement: Supplementary Information [file srep11467-s1.doc]

**Domain Walls Conductivity in Hybrid Organometallic Perovskites and Their Essential Role in CH_3_NH_3_PbI_3_ Solar Cell High Performance**

**(Supplementary Information)**

Sergey N. Rashkeev^1,*^, Fedwa El-Mellouhi^1^, Sabre Kais^1,2^, and Fahhad H. Alharbi^1^

^1^Qatar Foundation, Qatar Environment and Energy Research Institute, P. O. Box 5825, Doha, Qatar

^2^Department of Chemistry, Birck Nanotechnology Center, Purdue University, West Lafayette, IN 47907, USA

*email: [srashkeev@qf.org.qa](mailto:srashkeev@qf.org.qa)

1. **LGD theory for the Domain Walls**

Following Ref. [S1], let us first consider a head-to-head and tail-to-tail inclined wall in a uniaxial ferroelectric semiconductor doped with *n*-type impurity (for *p*- type doping the results are similar and will be discussed below). A sketch of the charged walls is shown in Fig. S1. Here *θ* is the incline angle of the domain wall (the angle between the wall plane and the polarization vector of the uniaxial ferroelectric), the normal vectors to both film interfaces with electron and hole conductors are oriented along z axis. We suggest that the domain wall is planar as it can be approximated for reasonably very small segment of any wall. For the uniaxial ferroelectrics, the electric field potential *φ*(*x,z*) and the ferroelectric polarization component *P_z_*(*x,z*) should be determined from the Poisson equation.

$\varepsilon_{0}\left(\varepsilon_{33}^{b}\frac{\partial^{2}\phi}{\partial z^{2}}+\varepsilon_{11}\frac{\partial^{2}\phi}{\partial x^{2}}\right)=\frac{\partial P_{z}}{\partial z}-q\cdot\left[N_{d}^{+}(\phi)+p(\phi)-n(\phi)-N_{a}^{-}\right]$ , (S.1)

with the boundary conditions of the potential vanishing far from the domain wall,

$\phi(r\to\pm\infty)=0$ , (S.2)

where *q* = 1.6×10^−19^ C is the electron charge, *ε*_0_ = 8.85×10^−12^ F/m – the universal dielectric constant, *ε*_11_ is the dielectric permittivity in the direction normal to the polar axis, and $\varepsilon_{33}^{b}$ is the background or base dielectric permittivity different from the ferroelectric soft-mode permittivity *ε*_33_, which is usually much lower than *ε*_33_*,* since its origin can be related to electronic polarizability from the nonferroelectric lattice modes of the crystal [S2]. For *n*- doped material, ionized deep acceptors with field-independent concentration $N_{a}^{-}$ play the role of a background charge; ionized shallow donors, free holes, and electron equilibrium concentration are $N_{d}^{+}$, *p*, and *n*, respectively. However, solar cell irradiated by light is not in thermal equilibrium, i.e., one should use different quasi-Fermi energies for electrons and holes, $E_{F}^{e}$ and $E_{F}^{p}$,


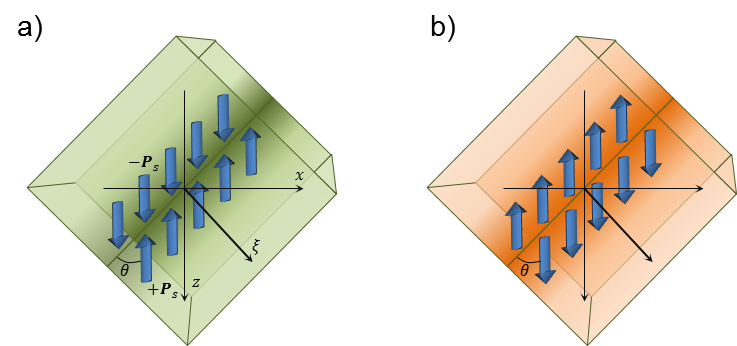


**Figure S1**. Sketch of the charged walls in the uniaxial ferroelectric *n*- type semiconductor film: (a) inclined head-to-head, (b) inclined tail-to-tail domain walls. Green (orange) gradient color corresponds to excess negative (positive) charge density at the domain-wall vicinity. For *n-* doped material, the excess negative charge is related to electrons while the excess positive charge – to both free-carrier holes and charged donor impurities (see the discussion below).

$\begin{matrix} E_{F}^{e}=E_{C}-k_{B}T\ln\left(\frac{N_{C}}{n_{\infty}}\right) \\ E_{F}^{p}=E_{V}+k_{B}T\ln\left(\frac{N_{V}}{p_{\infty}}\right) \end{matrix}$ , (S.3)

where *E_C_* is the bottom of the conduction band, *E_V_* is the top of the valence band, *k_B_* = 1.3807×10^-23^ J/K is the Boltzmann’s constant, *T* is the absolute temperature, and

, (S.4)

are the so-called effective densities of states at conduction band minimum and valence band maximum, *m_e_* and *m_p_* are the electron and hole effective masses, respectively. $n_{\infty}$and are the electron and hole concentration at large distances from any domain wall (bulk densities),

$\begin{matrix} p_{\infty}=G\cdot\tau\\ n_{\infty}=G\cdot\tau+N_{d}^{+}(\infty) \end{matrix}$ . (S.5)

Here *G* is the generation time of electron-hole pairs which depends on the intensity of light (solar) radiation, *τ* is the minority charge carrier recombination rate, $N_{d}^{+}(\infty)$ is the concentration of charged donor impurities in the bulk.

Then the concentration of the electrons in the conduction band and holes in the valence band is,

$\begin{matrix} n(\phi)=N_{C}\exp\left(\frac{-E_{C}+E_{F}^{e}+q\phi}{k_{B}T}\right) \\ p(\phi)=N_{V}\exp\left(\frac{E_{V}-E_{F}^{p}-q\phi}{k_{B}T}\right) \end{matrix}$ . (S.6)

If we consider the donor level as infinitely thin level with activation energy *E_d_*, the concentration of donors is determined as,

$N_{d}^{+}(\phi)=N_{d0}\cdot[1-f(E_{d}-(E_{F}^{e}+q\phi))]$ , (S.7)

where *N_d0_* is the concentration of donor centers in the semiconductor, *f* is the Fermi distribution function.

Eqs. (S.6) and (S.7) are written in the Boltzmann approximation which does not take into account any quantum effects. According to these formulas, the electron concentration grows exponentially with potential in the regions of positive potential. This is incorrect when the electron gas becomes degenerated, and the Thomas-Fermi formula for the electron density should be employed,

, (S.8)

which is valid in the vicinity of the domain walls.

Due to the potential vanishing far from the wall, the electroneutrality condition should be valid,

$N_{a}^{-}+n_{\infty}=N_{d}^{+}(\infty)+p_{\infty}$ . (S.9)

The *z*- component of the polarization vector satisfies the LGD equation,

$\alpha(T)P_{z}+\beta P_{z}^{3}+\gamma P_{z}^{5}-g\left(\frac{\partial^{2}P_{z}}{\partial z^{2}}+\frac{\partial^{2}P_{z}}{\partial x^{2}}\right)=-\frac{\partial\phi}{\partial z},$ (S.10)

where *α(T)*, *β*, and *γ* (*α(T) <* 0 at temperatures below 330 K corresponding to the temperature of the tetragonal to cubic phase transition, Ref. [S3]) are the materials parameters of the LGD phenomenological free energy, *g* is the coefficient for the gradient term which is related to the coherence length, *r_c_*,

$r_{c}=\sqrt{\frac{-g}{\alpha(T)}}$ . (S.11)

The boundary conditions for the ferroelectric order parameter, *P_z_*, are

$P_{z}(r\to\pm\infty)=\pm P_{S}$ , (S.12)

with *P_S_* being the spontaneous ferroelectric polarization. By introducing a new variable which characterizes a distance to the domain wall plane,

$\xi=x\cos\theta+z\sin\theta,$ (S.13)

the coupled system of LGD and Poisson equations may be rewritten as,

$\begin{matrix} \alpha(T)P_{z}+\beta P_{z}^{3}+\gamma P_{z}^{5}-g\frac{\partial^{2}P_{z}}{\partial\xi^{2}}=-\sin\theta\frac{\partial\phi}{\partial\xi} \\ \varepsilon_{0}\left(\varepsilon_{33}^{b}\sin^{2}\theta+\varepsilon_{11}\cos^{2}\theta\right)\frac{\partial^{2}\phi}{\partial\xi^{2}}=\sin\theta\frac{\partial P_{z}}{\partial\xi}-q\cdot\left[N_{d}^{+}(\phi)+p(\phi)-n(\phi)-N_{a}^{-}\right] \end{matrix}$ , (S.14)

with boundary conditions,

$\begin{matrix} P_{z}(\xi\to\pm\infty)=\pm P_{S} \\ \phi(\xi\to\pm\infty)=0 \end{matrix}$ . (S.15)

All ion contributions to the static conductance are neglected since the ion mobility is much smaller than the electron and hole ones. So the static conductance can be calculated as

$\sigma(\xi)=q\cdot[\mu_{e}n(\xi)+\mu_{p}p(\xi)]$, (S.16)

where *μ_e_* and *μ_p_* are the electron and hole mobilities.

**2. LGD Parameters for MAPbI_3_**

To perform numerical calculations for ferroelectric domain walls in real life organometallic materials, one needs to know a significant number of different parameters including dielectric function for different frequency ranges and different polarizations, effective masses, coherence length, spontaneous polarization, etc. Unfortunately, for most of the (RNH_3_)MX_3_ materials (R is an organic group, R=H-, CH_3_-, NH_3_CH-, etc.; M is Pb or Sn; and X is a halogen I, Br, or Cl), a complete set of such experimental and/or theoretical data is still missing or incomplete and sometimes contradictory. The most investigated compound is MAPbI_3_ which has been investigated for a long time starting 1980s. A review of previous measurements of dielectric function at different frequency regions were performed in Ref. [S4] and provides the values of $\varepsilon_{33}^{b}$= 6.5, *ε*_33_ = 32, and *ε*_11_ = 62, at room temperature.

The magnitude of the bulk polarization in MAPbI_3_ has been probed using Berry phase calculations within the modern theory of polarization using the PBEsol density functional [S5]. The electronic polarization of MAPbI_3_ was found to be *P_S_* = 38 μC/cm^2^ which is comparable to other inorganic ferroelectric oxide perovskites (e.g., *P_S_* ~ 30 μC/cm^2^ in KNbO_3_).

In other article, the authors explored the effect of molecular orientation on the structure and shift current and constructed two 2x2 supercell structures (called M1 and M2), starting from the tetragonal PbI_3_ inorganic frame [S6]. These structures are essentially different from the pristine tetragonal structure, and the polarization of each of them is different from that of tetragonal structure. In order to show that the polarization value does not significantly affect our main conclusions, we repeated our calculations for the value of *P_S_* = 5 μC/cm^2^ for M1 structure of MAPbI_3_ (Table 2 of Ref. [S6]) without changing any other parameters. As one could expect, the only significant change is the concentration of charged particles at the wall (and corresponding electrostatic potential in the vicinity of the wall) which is proportional to the polarization discontinuity step (which is obvious from the relation *div****P*** *= 4*
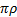
, where ***P*** is the polarization vector, *ρ* is the concentration of mobile charge carriers). The ratio of conductivity at the wall to the conductivity in the bulk is also proportional to this concentration. The value of this ratio is still very high even for *P_S_* = 5 μC/cm^2^, and our qualitative conclusions are still valid.

These set of data allows calculating the parameters of LGD functional. Coefficients *α* and *β* in Eqs. (S.10) and (S.14) could be obtained from the relations (see Ref. [S7] for details),

$\alpha=-\frac{1}{2\varepsilon_{0}\varepsilon_{33}}$ = -1.883×10^9^ m/F , (S.17)

and

$\beta=-\frac{\alpha}{P_{S}^{2}}$ = 1.304×10^10^ m^5^C^-2^F^-1^ , (S.18)

for *P_S_* = 38 μC/cm^2^. The coercive field *E_c_* defined as the turning point$\left(dE/dP\right)_{E=E_{c}}=0$, could be calculated as

$E_{c}=\pm2\sqrt{\frac{-\alpha^{3}}{27\beta}}=\pm\frac{2}{3\sqrt{3}}P_{S}\left|\alpha\right|=\pm$2.754×10^8^ V/m . (S.19)

We also assume that we have a second order ferroelectric phase transition, i.e., the LGD functional parameter *γ* is zero. The correlation length for the structure of MAPbI_3_ within mesoporous titania has been investigated using X-ray scattering [S8]. It was found that the majority of the MAPbI_3_ exists in a disordered state with a structural coherence length of only *r_c_* = 14 Å, i.e., the length of just two PbI_3_ cages only, which gives *g* = 3.691×10^-9^ m^3^/F (see Eq.(S.11)).

We also use the values of effective masses calculated by using an approach which includes both the spin-orbit coupling and GW method which are *m_e_*=0.16·*m* and *m_p_*=0.32·*m* (*m* is the electron mass), for the electron and hole effective masses averaged over all directions in Brillouin zone in MAPbI_3_ crystalline material [S9].

A knowledge of the linear absorption coefficient of MAPbI_3_ (5.7×10^4^ cm^−1^ at 600 nm) and transient absorption time decay (~5.6 ns) [S10], allows estimate the electron-hole pair generation rate. If we take the value of 4.8×10^-2^ J/(cm^2^·s) = 3×10^17^ eV/(cm^2^·s) for daylight solar power flux, suggest that each photon creates only one electron-hole pair, and assume that the average energy of the photon in solar spectrum is ~2 eV, we get Φ ~ 1.5×10^17^ photons/(cm^2^·s) for the solar photon flux Φ, so the electron-hole pair generation rate is

$G=\Phi\cdot\alpha$= 9.38×10^21^ cm^-3^·s^-1^ $\approx$10^22^ cm^-3^·s^-1^ . (S.20)

Now it is possible to estimate typical steady-state concentrations of the electron and hole charge carriers injected in MAPbI_3_. Using the value of the electron-hole pair generation rate for a daytime sunlight power (Eq.(S.20)), and charge carrier lifetime obtained from transient absorption time decay [S10], we find that typical concentrations of charge carriers injected by solar light do not exceed 10^13^ – 10^14^ cm^-3^ which is lower than typical concentrations of impurities in doped semiconductors. It means that all dopants which could be introduced or formed in the film during the preparation process should be taken into account because the contribution of these defects and impurities to the concentration of the charge carriers (and, therefore, the conductance) could be much higher than the contribution of solar injected charge carriers.

**3, Head-to-head domain walls**

The polarization vector, electric field, electric field potential *φ*(ξ), and concentration of electrons *n*(ξ) and charged donors *N_d_^+^*(ξ) as a function of the distance from the wall plane, ξ (measured in the units of the correlation radius, *r_c_*), calculated for the inclined head-to-head domain walls with different slope angles *θ* and two different concentrations of donor impurities are shown in Figures S2 and S3. The uncharged wall (*θ* = 0) is the thinnest; the charged perpendicular wall (*θ* = π/2) with maximal bound charge is the thickest. Correspondingly, the electric field potential created by the wall bound charges and screening carriers is the highest for the perpendicular wall (*θ* = π/2) with maximal bound charge 2*P_S_*. It decreases with the bound charge decrease (*θ* decrease), since the bound charge is 2*P_S_* ·sin*θ*, and vanishes at *θ* = 0. The net electric field of the bound charge attracts free electrons (in the accumulation region with |ξ| < 15·*r_c_*, see Figs. S2(c), S3(c)). The electron concentration is also the highest for the perpendicular wall (*θ* = π/2); it decreases with the bound charge decrease (*θ* decrease) and vanishes at *θ* = 0.

The net electric field “repulses” ionized donors (neutralizes them in the region with excess concentrations of electrons) and forms ionized donor depletion region at distances |ξ| < 15·*r_c_* from the charged wall region (Figs. S2(d) and S3(d)). We introduced donor impurities in the model because the structure and chemistry of thin perovskite films is extremely dependent on different details of the preparation process. In this process, many different charged defects could be introduced and play a role of dopants in this semiconductor (see Ref. [S3] for more discussion). First, we consider charged domain walls in *n*- doped ferroelectric semiconductor in which the electronic conductance should dominate (the p- doped materials will be discussed below).


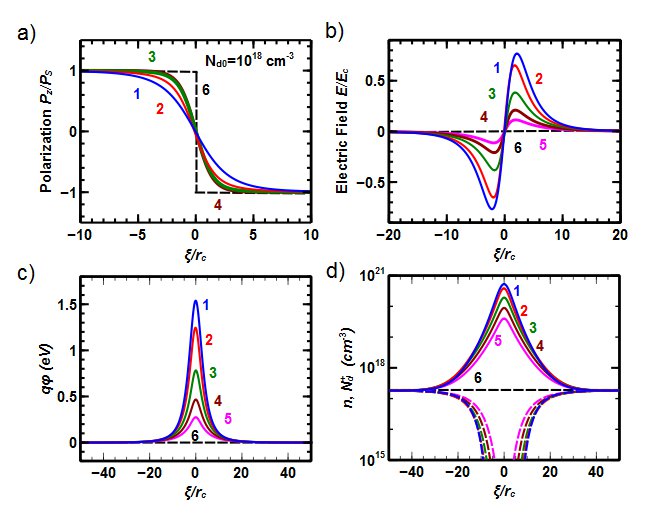


**Figure S2**. Dependencies of: (a) polarization *P_z_(*ξ*)/P_S_*; (b) electric field *E/E_c_*; (c) potential energy *qφ(*ξ*)*; (d) concentrations of electrons (solid lines) and ionized donors (dashed lines), in the vicinity of the inclined head-to-head domain wall with different incline angles *θ* = *π*/2; *π*/4; *π*/8; *π*/16; *π*/32; 0 (curves 1 – 6), ξ is measured in the units of the coherence radius *r_c_*. The concentration of *n* donors in the bulk of material is *N_d0_* = 10^18^ cm^-3^.


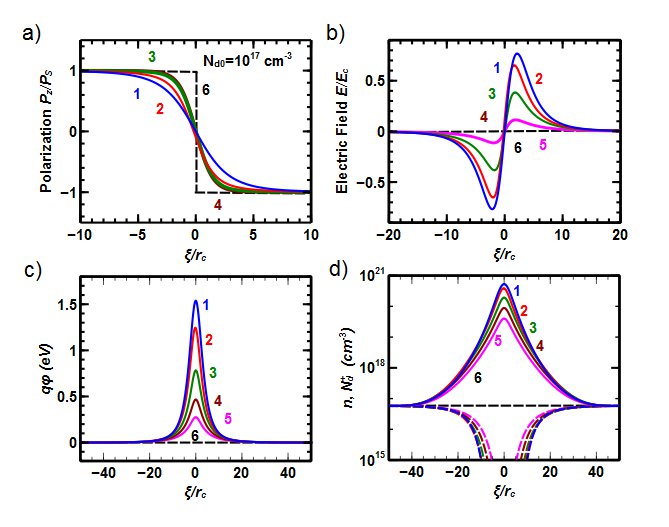


**Figure S3**. Dependencies of: (a) polarization *P_z_(*ξ*)/P_S_*; (b) electric field *E/E_c_*; (c) potential energy *qφ(*ξ*)*; (d) concentrations of electrons (solid lines) and ionized donors (dashed lines), in the vicinity of the inclined head-to-head domain wall with different incline angles *θ* = *π*/2; *π*/4; *π*/8; *π*/16; *π*/32; 0 (curves 1 – 6); ξ is measured in the units of the coherence radius *r_c_*. The concentration of *n* donors in the bulk of material is *N_d0_* = 10^17^ cm^-3^.

Now we can compare the characteristics of the domain walls at different doping levels. From Figs. S2(c) and S3(c), it is apparent that the electric field in the vicinity of the wall does not depend on the doping level. The wall accumulates electrons from nearby region in order to compensate the effects of the bound charges at the domain wall (and the polarization field discontinuity). The electron attraction to this region naturally stops when the electron concentration reaches a saturation level which depends only on the value of spontaneous polarization field, not on the average electron charge carrier concentration. Because of this reason, the value of electron concentration at ξ = 0 is nearly the same for both dopant concentrations (Figs. S2(d) and S3(d)). However, the decline of the charge carrier concentration with the increase of |ξ| is different. For every case, the concentration steadily approaches its bulk value when moving away from the domain wall. For the head-to-head domain walls and *n*- doped material, the concentration of charged donors is depleted in the wall region because the probability for a charged donor defect to become neutral increases with the increase of concentration of free electrons. The main difference between the curves plotted for two different concentrations of *n* donors are different values of $n_{\infty}$and $N_{d}^{+}(\infty)$ - the saturated values of electrons and charged donor impurities at large distance from the domain wall. These two values are nearly equal at range of considered donor impurity concentrations because the concentration of light injected holes is much lower (*p* ~ 10^13^ – 10^14^ cm^-3^). Calculations also indicated that the holes concentration in the vicinity of the wall is much lower than the concentration of electrons (Figure S4(a)), i.e., one can neglect hole related conductivity at the head-to-head domain wall.

As a result of electron accumulation near the head-to-head domain wall, the static conductivity drastically increases at the wall – up to 2 – 3 orders of magnitude for the dopant level of *N_d0_* = 10^18^ cm^-3^ (Figure S4(b)). Also, conductance in the direction parallel to the wall is maximal for a perpendicular wall (*θ* = π/2) and is zero for the wall with the plane perpendicular to the surfaces of ferroelectric film (*θ* = 0). Discussing the applicability of the film for solar cell development, one should be interested in the electric current flowing in the direction perpendicular to the film surfaces, which may be achieved only in domain walls inclined with angle *θ* different from π/2 (in which case the current flows parallel to the film surface and does not contribute to the photocurrent). Also, for angle *θ* = 0, there is no current across the film because this domain wall does not accumulate any charge. For angles different from 0 and π/2, one should multiply the current density at the domain wall by cos*θ* to find the current component perpendicular to the film. Also, the resistance of the domain wall which propagates across the whole film is proportional to the length of the path of the charge carrier, i.e., to 1/cos*θ*, and this factor should be also taken into account in the solar cell design.


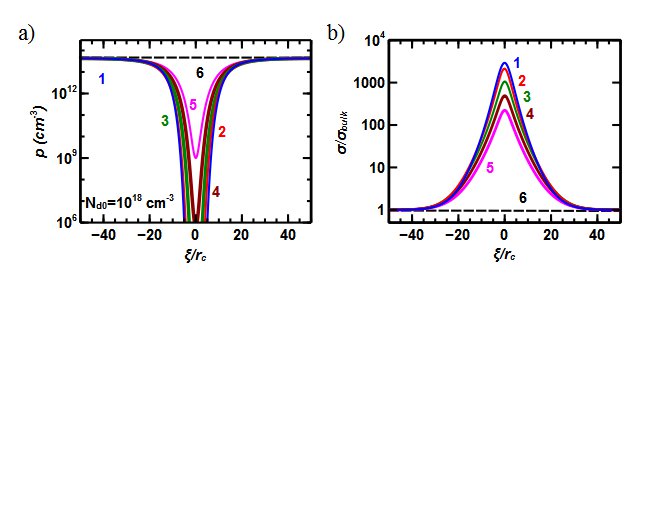


**Figure S4**. (a) Concentration of hole carriers, and; (b) the value of local conductance to bulk conductance ratio, as functions of ξ/*r_c_* in the vicinity of the inclined head-to-head domain wall with different incline angles *θ* = *π*/2; *π*/4; *π*/8; *π*/16; *π*/32; 0 (curves 1 – 6). The concentration of *n* donors in the bulk of material is *N_d0_* = 10^18^ cm^-3^.


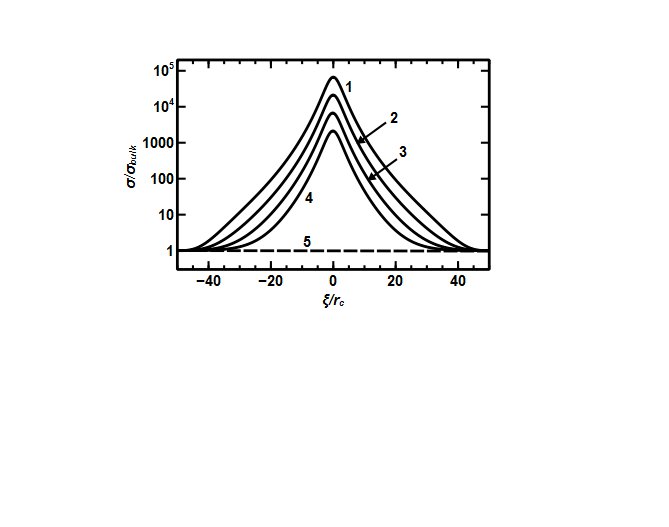


**Figure S5**. The value of local conductance to bulk conductance ratio, as functions of ξ/*r_c_* in the vicinity of the inclined head-to-head domain wall with incline angle *θ* = *π*/4 and different concentration of *n* donors in the bulk of material *N_d0_* = 10^15^ ; 10^16^; 10^17^; 10^18^; 0 cm^-3^ (curves 1 – 5).

Figure S5 shows the local-to-bulk conduction ratio, σ/σ_bulk_, at the head-to-head domain wall for incline angle *Θ* = π/4 and different dopant concentrations. It is apparent that the ratio goes up for smaller *N_d0_* reaching the value of ~10^5^ for *N_d0_* = 10^15^ cm^-3^. Again, such a behavior is not surprising because the “saturated” value of the electron concentration at the wall does not depend on *N_d0_* while the electron concentration of the bulk is completely defined by *N_d0_* and the position of the dopant impurity level relative to the conduction band edge (and *n* is smaller in the system with smaller *N_d0_*).

**4. Tail-to-tail domain walls**


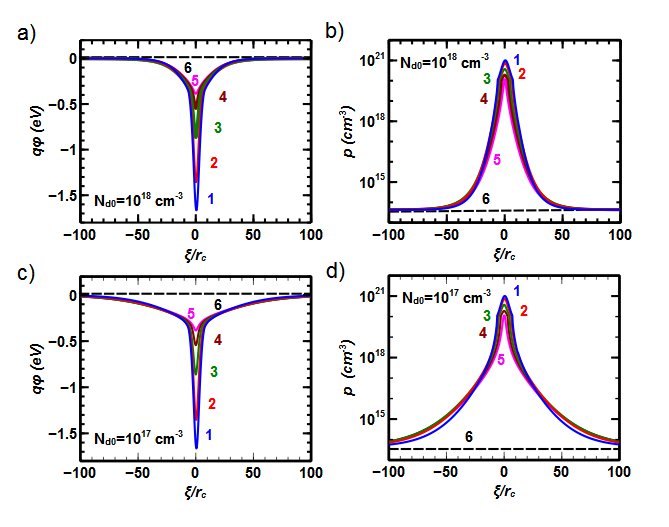


**Figure S6**. (a) Potential *φ(*ξ*)* for the concentration of *n* donors in the bulk *N_d0_* = 10^18^ cm^-3^; (b) concentration of holes *p(*ξ*)* for *N_d0_* = 10^18^ cm^-3^; (c) *φ(*ξ*)* for *N_d0_* = 10^17^ cm^-3^; (d) *p(*ξ*)* for *N_d0_* = 10^17^ cm^-3^, in the vicinity of the inclined tail-to-tail domain wall with different incline angles *θ* = *π*/2; *π*/4; *π*/8; *π*/16; *π*/32; 0 (curves 1 – 6). ξ is measured in the units of the coherence radius *r_c_*.

Calculations the tail-to-tail walls in *n*- doped semiconductors are similar. Namely, the electric field potential at the tail-to-tail wall shows a very narrow and deep peak at |ξ| < 10·*r_c_* surrounded by a smooth, slowly growing “background” with the width strongly depending on *N_d0_* (being |ξ| < 50·*r_c_* for *N_d0_* = 10^18^ cm^-3^ and |ξ| < 120·*r_c_* for *N_d0_* = 10^17^ cm^-3^; Figs. S6(a) and S6(c)). The sharp dip corresponds mainly to degenerated holes gas near the wall while the background is related to slow decay of the hole concentration to the saturated hole density at large distances from the wall which is several orders of magnitude lower than saturated electron density in *n*- doped semiconductor (Figs. S6(b) and S6(d)). Also, the thicker background layer contains accumulated positively charged donors and exhibits electron depletion. Such a behavior is due to electric field potential that pushes electrons away from both the dip and background regions thus making appearance of positively charged donor impurities more favorable than in the bulk of the semiconductor (Figure S7).


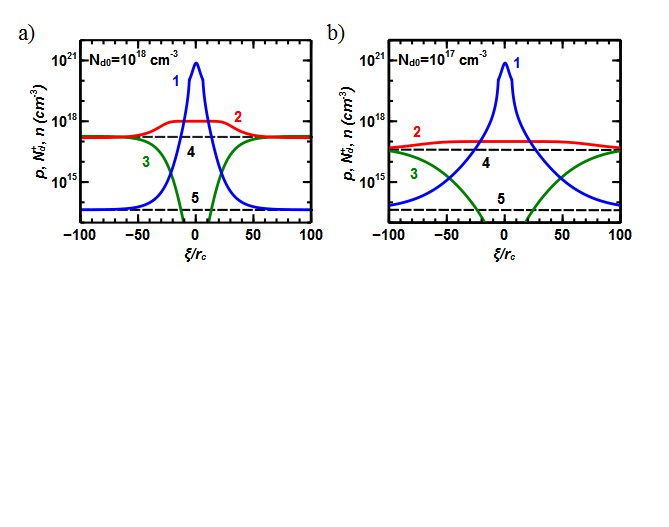


**Figure S7**. Dependencies of the hole density, *p(*ξ*)* (blue curves 1); positively charged donor density $N_{d}^{+}$ (red curves 2), and; electron density, *n(*ξ*)* (green curves 3) for the concentration of *n* donors in the bulk: (a) *N_d0_* = 10^18^ cm^-3^ ; (b) *N_d0_* = 10^17^ cm^-3^. The incline angle is *θ* = *π*/4. ξ is measured in the units of the coherence radius *r_c_*. Dashed lines 4 and 5 correspond to saturated values of the electron and hole densities at large distance from the tail-to-tail domain wall.

Although there are differences in the behavior of positively (tail-to-tail) and negatively (head-to-head) charged domain walls, the qualitative description of the conductance mechanism near the walls are very similar because for both types of walls the highest charge carrier transport occurs mainly in very thin layer near the wall. Also, each wall conducts only one type of charge carriers (electrons or holes), i.e., a significant spatial separation of charge carriers of different types will take place which should reduce the recombination of the mobile carriers. In particular, holes accumulation near the tail-to-tail domain wall drastically increases the static conductance at the wall (up to 4 orders of magnitude for dopant level of *N_d0_* = 10^17^–10^18^ cm^-3^; Figure S8). Also, conductance in the direction parallel to the wall is maximal for a perpendicular wall (*θ* = π/2). There is, however, an important difference between the head-to-head and tail-to-tail walls in *n*- doped semiconductors. At the head-to-head wall, the local-to-bulk conduction ratio is continuously declining from its maximum at the wall to its bulk value of unity (Figures S4(b) and S5). This happens because the holes (minority carriers) contribution in the conductance is small compared to the electron contribution in all system. At the tail-to-tail wall, the concentration of minority carriers (holes) significantly exceeds the concentration of majority carriers (electrons) at the vicinity of the wall. Therefore, holes conductance takes place only near the wall (in the range |ξ| < 10·*r_c_*). The bulk (electron) conductance regime is reached at much larger distances (|ξ| > 50 – 100·*r_c_*) from the wall. Therefore, one can say that tail-to-tail wall with high holes conductance is separated (“insulated”) from the bulk material with electron conductance by a rather thick electron depleted layer. Such insulation should significantly reduce the possibility of charge carrier recombination at the tail-to-tail wall even further.

Before now, we considered the case of *n* doped semiconductor. The case of *p* doped material should be considered in similar way by permuting electron and hole densities and taking into account the concentration of negatively charged acceptor impurities instead of $N_{d}^{+}$ considered above. It such case, head-to-head (tail-to-tail) walls are becoming holes (electrons) conductors. The difference between the majority and minority carriers are also similar to the case of just considered *n* doped material.


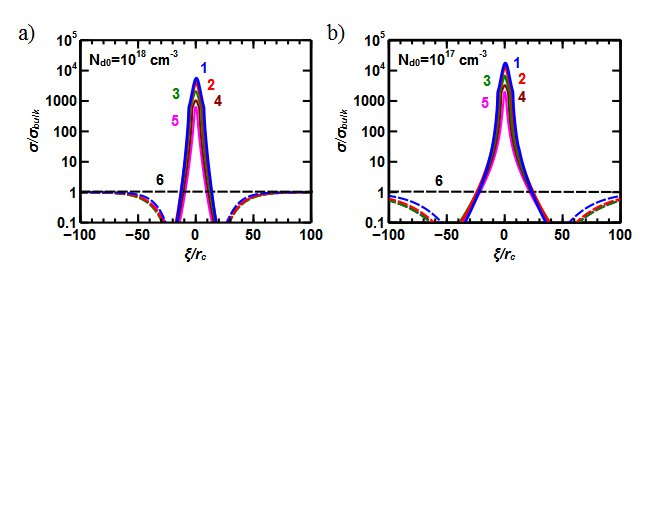


**Figure S8**. The values of local conductance to bulk (electronic) conductance ratio, as functions of ξ/*r_c_*, in the vicinity of the inclined tail-to-tail domain wall with the concentration of *n* donors in the bulk of material: (a) *N_d0_* = 10^18^ cm^-3^; (b) 0 *N_d0_* = 10^17^ cm^-3^. The incline angles are *θ* = *π*/2; *π*/4; *π*/8; *π*/16; *π*/32; 0. Solid lines 1 – 6 relate to the holes conductance at these values of angles; corresponding electron contributions for same angles are shown in dashed lines of the same color. ξ is measured in the units of the coherence radius *r_c_*.

We found that domain walls in MAPbI_3_ may indeed be responsible for the observed conductance and mean free path for charge carriers. Although it is difficult to provide an exact number for the domain wall related conductance in the perovskite film, it is apparent that it could be much higher than regular conductance mechanism through the bulk of semiconductor. Also, domain walls help separate electron and hole charge carriers thus reducing their recombination and increasing their lifetime.

**5. Mobility at the Domain Walls**

The electronic structure within the confinement can be calculated by solving the Schroedinger equation for the plane-perpendicular direction in the electrostatic potential *φ* shown in Figures S2 and S6 for the head-to-head and tail-to-tail domain walls. The periodic Bloch function cannot be employed in the direction of the confinement. Ideally, the electrostatic potential, wavefunctions, and the subband energy levels in this direction should be obtained by solving the Schroedinger, LGD, and Poisson equations self-consistently. However, for qualitative understanding of the subband energy levels, we may simply solve the Schroedinger equation for an electron in the potential *φ*. Assuming the quadratic form of the potential near the bottom, we find that the energy distance between the nearest transverse levels of resulting harmonic oscillator is 0.43 eV for *θ* = π/2 and 0.08 eV for *θ* = π/32, for the head-to-head wall which means that the dip of the potential energy in the plane-perpendicular direction for each incline angle should contain ~3 – 4 harmonic oscillator energy levels before reaching the continuum. For qualitative consideration, one could start with considering only one plane-perpendicular electronic subband and neglect possible multiband effects for both head-to-head and tail-to-tail walls.

In general, to evaluate the charge carrier (electrons (holes) for head-to-head (tail-to-tail) walls) mobility at the domain wall, one should consider several scattering mechanisms including: (i) phonon related intravalley scattering; (ii) phonon related intervalley g-process scattering; (iii) remote ionized impurity scattering, and; (iv) effects of possible interface (domain wall) roughness [S11,S12].

At low temperatures, the last two contributions should dominate. Here we concentrate mainly on scattering by remote ionized impurities. As we discussed above, the concentration of positive donor impurities is low in the vicinity of the head-to-head wall (Figures 2(d) and 3(d)). Therefore, electrons flowing along the domain wall feel only the electrostatic field of remote positively charge donor impurities positioned near the edges of the conductive channel (|ξ| ~ 15*r_c_*) or even further. The effects of these impurities should be similar to that in strained Si quantum well where the antimony (Sb) doped layer is positioned at 20 nm away from the strained Si channel [S12]. In the Born approximation, transport relaxation time related to intrasubband scattering is described by [S11],

, (S.21)

where *m_t_* is the transverse effective mass, is the two-dimensional energy spectrum related to motion of electron parallel to the domain wall, *θ* is the scattering angle between the incident and scattered vectors $\vec{k}$and $\vec{k}'$, $q=k\sqrt{2\cdot\left(1-\cos\theta\right)}$ is the momentum transfer, *z* is the distance between the ionized impurity and the domain wall, and the factor$\text{v}_{\vec{q}}(z)$is the effective impurity potential for electrons with dielectric screening (see Ref. [S11]),

, (S.22)

where $\bar{\varepsilon}$ is an arithmetic average of the static dielectric functions of materials positioned at the both sides of the interface (in this case, domain wall), and the exponential screening corresponds to the screened Coulomb potential of a single impurity positioned at the distance *z* from the interface.

We also assume that the charged centers are distributed completely at random in the plane parallel to the wall. The mobility is given by

$\mu=\frac{e}{m_{t}}\left\langle\tau\right\rangle$ , (S.23)

with

, (S.24)

where $f((\varepsilon(\vec{k}))$is the Fermi distribution function. At sufficiency low temperatures, $\left\langle\tau\right\rangle=\tau(E_{F})$, with *E_F_* being the Fermi energy of the two-dimensional electron gas,

, (S.25)

and the Fermi wavevector is

$k_{F}=\left(2\pi N_{s}/g_{v}\right)^{1/2}$ . (S.26)

Here *N_s_* is the two-dimensional concentration of electrons in the accumulation layer,

$N_{s}=r_{c}\int_{-\infty}^{\infty} n\left(y\right)\cdot dy$ , (S.27)

with *y* = ξ/*r_c_*; *g_v_* is the valley degeneration factor (see Ref. [S11]).

Simplifying Eq. (S.21) one gets,

, (S.28)

where *m* is a free electron mass.

Although Eq. (S.28) was derived for the head-to-head domain wall, it is quite general and can be used for the tail-to-tail walls as well. In this case, the parameters *m_t_*, *N_s_*, and *k_F_* should be the transverse hole effective mass, two-dimensional density of holes at the wall, and the corresponding Fermi wavevector. The scattering by remote ionized impurities, however, should be much stronger for holes at the tail-to-tail domain walls than for electrons at the head-to-head wall because the concentration of ionized impurities at the tail-to-tail wall has a maximum (not minimum as for the head-to-head walls; see Fig. S7). Moreover, this concentration is even higher than the equilibrium charged donor impurity concentration at large distances from the wall. Therefore, the ionized impurities cannot even be called “remote” anymore – they are present in the region with high holes concentration and the corresponding hole scattering should be higher than electron scattering at the head-to-head walls.

**References**:

[S1] Eliseev, E. A., Morozovska, A. N., Svechnikov, G. S., Gopalan, V. & Shur, V. Ya. Static conductivity of charged domain walls in uniaxial ferroelectric semiconductors. *Phys. Rev. B* **83**, 235313 (2011).

[S2] Tagantsev, A. K. & Gerra, G. Interface-induced phenomena in polarization response of ferroelectric thin films. *J. Appl. Phys.* **100**, 051607 (2006).

[S3] Stoumpos, C. C., Malliakas, C. D. & Kanatzidis, M. G. Semiconducting tin and lead iodide perovskites with organic cations: Phase transitions, high mobilities, and near-infrared photoluminescent properties. *Inorg. Chem*. **52**, 9019−9038 (2013).

[S4] Even, J., Pedesseau, L. & Katan, C. Analysis of multivalley and multibandgap absorption and enhancement of free carriers related to exciton screening in hybrid perovskites. *J. Phys. Chem. Lett*. **4**, 2999−3005 (2013).

[S5] Frost, J. M., Butler, K. T., Brivio, F., Hendon, C. H., Schilfgaarde, M. & Walsh, A. Atomistic origins of high-performance in hybrid halide perovskite solar cells. *Nano Lett*. **14**, 2584−2590 (2014).

[S6] Zheng, F., Takenaka, H., Wang, F., Koocher, N. Z. & Rappe, A. M. First-principles calculation of bulk photovoltaic effect in CH_3_NH_3_PbI_3_ and CH_3_NH_3_PbI_3−_*_x_*Cl*_x_*. e-print arXiv:1410.3389v1.

[S7] Kim, S., Gopalan, V. & Gruverman, A. Coercive fields in ferroelectrics: A case study in lithium niobate and lithium tantalite. *Appl. Phys. Lett*. **80**, 2740-2742 (2002).

[S8] Choi, J. J., Yang, X., Norman, Z. M., Billinge, S. J. & Owen, J. S. Structure of methylammonium lead iodide within mesoporous titanium dioxide: active material in high-performance perovskite solar cells. *Nano Lett*. **14**, 127–133 (2014).

[S9] Umari, P., Mosconi, E. & De Angelis, F. Relativistic GW calculations on CH_3_NH_3_PbI_3_ and CH_3_NH_3_SnI_3_ perovskites for solar cell applications. *Scientific Reports* **4**, 4467 (2014).

[S10] Xing, G. *et al.* Long-range balanced electron and hole-transport lengths in organic-inorganic CH_3_NH_3_PbI_3_. *Science* **342**, 344−347 (2013).

[S11] Ando, T., Alan B. Fowler, A, B, & Stern, F. Electronic properties of two-dimensional systems. *Rev. Mod. Phys.* **54**, 437-672 (1982).

[S12] Tanaka, T. *et al*. Experimental and theoretical analysis of the temperature dependence of the two-dimensional electron mobility in a strained Si quantum well. *J. Appl. Phys*. **111**, 073715 (2012).
